# Assessment of demographic and perinatal predictors of non-response and impact of non-response on measures of association in a population-based case control study: findings from the Georgia Study to Explore Early Development

**DOI:** 10.1186/s12982-018-0081-y

**Published:** 2018-08-16

**Authors:** Laura A. Schieve, Shericka Harris, Matthew J. Maenner, Aimee Alexander, Nicole F. Dowling

**Affiliations:** National Center on Birth Defects and Developmental Disabilities, Centers for Disease Control and Prevention, Mailstop E-86, 4770 Buford Hwy NE, Atlanta, GA 30341 USA

**Keywords:** Epidemiologic research design, Reproducibility of results, Data accuracy, Autism spectrum disorder, Risk factor, Case–control study, Selection bias

## Abstract

**Background:**

Participation in epidemiologic studies has declined, raising concerns about selection bias. While estimates derived from epidemiologic studies have been shown to be robust under a wide range of scenarios, additional empiric study is needed. The Georgia Study to Explore Early Development (GA SEED), a population-based case–control study of risk factors for autism spectrum disorder (ASD), provided an opportunity to explore factors associated with non-participation and potential impacts of non-participation on association studies.

**Methods:**

GA SEED recruited preschool-aged children residing in metropolitan-Atlanta during 2007–2012. Children with ASD were identified from multiple schools and healthcare providers serving children with disabilities; children from the general population (POP) were randomly sampled from birth records. Recruitment was via mailed invitation letter with follow-up phone calls. Eligibility criteria included birth and current residence in study area and an English-speaking caregiver. Many children identified for potential inclusion could not be contacted. We used data from birth certificates to examine demographic and perinatal factors associated with participation in GA SEED and completion of the data collection protocol. We also compared ASD-risk factor associations for the final sample of children who completed the study with the initial sample of all likely ASD and POP children invited to potentially participate in the study, had they been eligible. Finally, we derived post-stratification sampling weights for participants who completed the study and compared weighted and unweighted associations between ASD and two factors collected via post-enrollment maternal interview: infertility and reproductive stoppage.

**Results:**

Maternal age and education were independently associated with participation in the POP group. Maternal education was independently associated with participation in the ASD group. Numerous other demographic and perinatal factors were not associated with participation. Moreover, unadjusted and adjusted odds ratios for associations between ASD and several demographic and perinatal factors were similar between the final sample of study completers and the total invited sample. Odds ratios for associations between ASD and infertility and reproductive stoppage were also similar in unweighted and weighted analyses of the study completion sample.

**Conclusions:**

These findings suggest that effect estimates from SEED risk factor analyses, particularly those of non-demographic factors, are likely robust.

## Background

Observational epidemiologic studies are vital to the development of the knowledge base characterizing risk and preventive factors for health conditions and disabilities. However, the findings from these studies must be interpreted with careful consideration of any threats to internal validity, such as missing data due to non-participation. Over the past several decades, participation rates in epidemiologic studies have steadily declined, raising concern that the findings from these studies might be increasingly influenced by potential selection bias [1–3]. However, the estimates derived from epidemiologic studies have been shown to be robust under a wide range of scenarios [4]. In general, a high level of non-participation is, by itself, not sufficient to bias estimates. Odds ratios and other effect estimates from complete records analyses are asymptotically unbiased estimates, provided that missingness is not associated with *both* the exposure (or more generally the primary health factor under investigation) and the outcome variable [2, 4]. While empiric assessments of non-response are inherently challenging, given the lack of information available on non-responders in most studies, several studies with data to model non-response impacts on prevalence estimates from population-based surveys [2, 5–7] and effect estimates from analytic epidemiologic studies [8–13] have been reassuring. However, other studies have demonstrated that estimates from surveys with low response rates may be biased, particularly if the indicator of interest is a socio-demographic factor or a factor that is highly variable across population socio-demographic subgroups, such as certain risky health behaviors [14–16]. Factors reported to be associated with non-response in various studies include demographic factors such as low education [5, 9, 10, 16], male sex [5, 7, 8, 16], minority race-ethnicity [14], residence in area with socioeconomic deprivation [15], both younger age [7, 14, 16] and older age [5, 8], and increased number of children [11]. Health behaviors such as smoking and drinking have also been found to be associated with non-response in some studies [10, 11, 15, 16]. Nonetheless, in several studies, numerous specific health conditions and symptoms [5, 7, 11] were not associated response rates.

Additional assessments are needed to understand the effects of non-response, particularly from large population-based studies of disease/disability-risk factor associations that may be especially susceptible to differential non-participation in certain demographic subgroups.

The Georgia Study to Explore Early Development (GA SEED), a population-based case–control study designed to assess pre-conception and prenatal risk factors for autism spectrum disorder (ASD), provides a unique opportunity to explore factors associated with non-participation and the potential impact of non-response on odds ratios for associations between ASD and risk factors. GA SEED is one of six sites included in SEED, one of the largest multi-site studies of ASD risk factors to date. It was designed to include children and their caregivers from a range of sociodemographic and geographic subgroups, including many underserved and understudied population subgroups. This objective necessitated a complex study design that included identification of potential case children from multiple school and clinical sources throughout each site’s population-based area and identification of potential control children through random sampling of birth certificate files. While researchers utilized tracing procedures to locate hard-to-find participants, it was challenging to locate and determine SEED eligibility for many selected individuals, given the fairly high residential mobility rates of the US population, in general, and women in the child-bearing age range, in particular [17, 18], and the increasing move to cell phones instead of landlines, particularly for young adults [19].

While all SEED sites had access to limited analytic data from birth certificates for all enrolled participants, GA SEED additionally had access to these data on all individuals initially invited as potential participants. We used these data to compare our final participants who enrolled and completed the SEED protocol to individuals who were invited but not contacted, contacted but not enrolled, or enrolled but who dropped out before completing the study. We also used these data to develop sampling weights for our final complete sample of cases and controls in order to assess potential bias in our estimation of odds ratios for case–control comparisons of select risk factors.

## Methods

### Overview of SEED

GA SEED, along with five other population-based sites, followed the common SEED protocol [20]. Recruitment was focused on identification and enrollment of children for three study groups—children with ASD (ASD case group), children with other non-ASD developmental disabilities (DD group), and children without ASD from the general population (POP group). POP children were selected from random samples of the birth certificates within a given site’s defined geographic study area. Children for the ASD and DD groups were identified from multiple special education and clinical sources at each site that provide services to children with disabilities. To ensure that cases and POP controls were from the same study base, our sources for the ASD and DD groups included all major public school special education programs and the largest clinical sources serving children with ASD in the study area at the time of this study; thus, had they been identified as having ASD, children sampled for the POP group would have very likely been served at one of our data sources. Additionally, since POP controls were identified from birth certificates and ASD and DD children were identified from lists of pre-school aged children, to be eligible for this study, we required that all children had to have been both born and currently residing in the study area. We were intentionally broad in the types of disabilities included in our lists of children potentially eligible for the DD group. We focused on disabilities that are often seen as precursor or co-occurring diagnoses in children with ASD. In addition to serving as a second case group to understand similarities and differences between risk factors for ASD and other DDs, the DD group served as a means of finding yet undiagnosed ASD cases in young children who had come to the attention of a healthcare provider or school as having a developmental delay (see below). The GA SEED study site consisted of five metropolitan Atlanta-area counties.

Although children were initially identified as potentially being eligible for a given study group—ASD, DD, or POP—the final study group classification was determined from standardized research developmental assessments. Upon enrollment, all children were screened for possible autism characteristics through their caregiver’s completion of the Social Communication Questionnaire (SCQ). Children with SCQ scores above a predetermined threshold (≥ 11) were designated as presumptive ASD cases, regardless of how they were initially identified. All children who had a previous ASD diagnosis or autism special education classification were also designated as presumptive ASD cases regardless of their SCQ scores. All children were seen in person as part of the study protocol and administered a developmental assessment. Children in the presumptive ASD group (and their caregivers) underwent a more comprehensive assessment than children in the other groups, which included administration of two autism-specific assessments used to determine final case classification—the Autism Diagnostic Observation Schedule (administered to children) and the Autism Diagnostic Interview revised (administered to caregivers). Thus, final study group classification of ASD, DD, or POP did not always match initial (presumptive) classification at the time a child was initially identified and invited into the study (see Tables 1 and 2).

**Table 1:** Status of children in GA SEED^a^ from invitation to enrollment

Presumptive study classification at time of invitation^b^	ASD	DD	POP
No. invited	274	1968	2608
Final status of invited			
No. unable to contact—*but indication that contact info may not have been valid* (*mailing returned to sender; phone number not found, disconnected*, *or invalid*; *phone call reveals phone number does not belong to desired participant*)	92	696	1310
No. unable to contact—*no response to mailing or follow*-*up phone calls and no indication that contact information was invalid*	24	179	278
No. contacted, but child had aged out of study by time contact was made, eligibility screen not administered	3	241	170
No. contacted, but refused eligibility screen	46	334	358
No. contacted, but determined ineligible	18	146	266
No. contacted, eligible, but refused participation after eligibility screen	0	55	0
No. contacted, eligible and enrolled	91	317	226
Among those contacted, eligible and enrolled			
No. with positive ASD screen—and thus moved to presumptive ASD protocol workflow^c^	N/A^d^	139	9
Final no. in each presumptive workflow	239	178	217

^a^Children born in multiple births or missing data for birth certificate analyses not included in table because they were excluded from this analysis

^b^Children from school and health sources were identified as potential ASD if they had a previous diagnosis of ASD or special education classification of autism; they were identified as potential DD if they had a previous diagnosis or special education classification indicating a non-ASD developmental disability or delay. Potential POP children were randomly sampled from birth certificates

^c^At enrollment parents of children in all groups were administered a brief ASD screen; 148 children originally identified as potential DD or potential POP had a positive score on the screen and were moved into a study work flow designated presumptive ASD. This group received a more intensive developmental assessment than other children, including administration of two autism-specific instruments

^d^All children identified as having a previous diagnosis of ASD remained in the presumptive ASD group (which received the full autism-specific developmental assessment) regardless of their autism screen results obtained at enrollment

**Table 2:** Status of children included in GA SEED^a^ from enrollment to final classification

Presumptive study classification at time of enrollment	ASD	DD	POP
Total no. enrolled	239	178	217
No. who did not complete study	58	23	58
No. who completed study	181	155	159
Among those who completed the study, final classification based on results of study-administered developmental assessments			
ASD	118		
DD	63	155	
POP			159

^a^Children born in multiple births or missing data for birth certificate analyses not included in table because they were excluded from this analysis

Recruitment, enrollment, and data collection activities for the first phase of SEED occurred from 2007–2012 when children were 2–5 years of age. The most recent residences and phone numbers of children potentially eligible for GA SEED were provided by the schools and clinics in the study area. Likewise, contact information was available for mothers of the random sample of children born in the study area. Prior to invitation, the source lists were de-duplicated with each other and tracing was conducted to determine if more recent contact information was available.

Recruitment of children’s mothers consisted of (1) mailed invitation packet and (2) follow-up recruitment phone calls. The invitation packet included a letter introducing the study, a study brochure that provided a brief overview of SEED participant activities, and a response card and pre-paid envelope that the invitee was asked to return, indicating interest or non-interest in future contact to learn more about the study. If the respondent did not contact the study site within 14 days of the invitation mailing and a valid phone number was available, study staff attempted to contact the participant with up to nine phone calls (at various times during the day and different days of the week); calls were spread out over two or more months. Invitation materials and procedures were the same for all study groups. We did not provide information about whether children were being recruited as potential cases or controls.

We were unable to contact some participants. In other cases, the participant was delayed in responding to phone messages such that the child was older than 68 months at the time of contact (the maximum age for a valid assessment on certain developmental instruments included in the study). For more than 80% of those not contacted, we were not able to obtain complete or valid contact information, even after sustained tracing efforts. This was indicated by mailing returned as undeliverable and/or phone number not found during tracing or most recent phone number found was disconnected or determined to be the wrong number.

Once contact was made with the mother/other caregiver, study staff requested permission to administer a brief eligibility screen. Eligible children were born between 2003 and 2006, lived in the GA SEED defined study area both at birth and at study enrollment, and lived with a caregiver since 6 months of age who could provide legal consent and was capable of communicating in English. Additionally, children with vision, hearing or motor deficits that precluded administration of in-person assessments were ineligible for participation.

For children sampled from birth records, the biological mother was always the invitee. For a small percentage of children (2%) identified from school or clinical sources, another person was the primary caregiver.

After enrollment, a wealth of data was collected from children in all study groups and their caregivers, including an extensive interview with the mother about family socio-demographics, her reproductive history, and her pregnancy with the index child. The final study step was the aforementioned in-person developmental evaluation. In GA SEED, limited analytic birth certificate data were also available for all children included on initial invitation lists, whether or not they enrolled.

See Tables 1 and 2 for a full description of enrollment and study completion statuses for children initially invited to participate in GA SEED.

### GA SEED participation rates

There are several challenges in accurately determining research response rates for SEED. Because eligibility criteria required both birth and current residence (at age 2–5 years) in the study area and required the primary caregiver to have lived with the child since 6 months of age, to have legal guardianship, and to be able to communicate in English, it is likely that it was more difficult to make contact with ineligible than eligible families. Indeed, as stated, we had some indication that available contact information might not be valid for > 80% of those never contacted. This might be an indication that the family had moved, possibly outside the study area. Even among families who were contacted and agreed to the eligibility screen, ineligibility rates were high—54% for potential POP children, 28% for potential DD children and 17% for potential ASD children. Two reasons accounted for almost all ineligibility—the family had moved outside the study area (63%) or the mother/caregiver was not proficient in English (32%). While we can only speculate on ineligibility rates among those never contacted, it is possible that they were much more likely to have moved or to lack English-language proficiency than those we did contact.

Because of the likely high proportion of ineligibles among those never contacted, from its inception this analysis was designed to determine differences between the final sample of cases and controls and the total sample of likely case and control participants invited who did NOT complete the study for any reason (never contacted, contacted but ineligible, contacted but refused participation, contacted and enrolled but dropped out before completing the study). The basic premise of the SEED design was that associations between ASD and most non-sociodemographic risk factors should not be influenced by the SEED eligibility criteria, i.e. that the findings from SEED analyses should be generalizable to the population at large.

### Study objectives of analytic sample

The objectives of this analysis were to (1) understand how the distributions of demographic and perinatal factors among mother–child pairs who completed the study and were classified as ASD cases compared to mother–child pairs initially invited to potentially participate in SEED who had an indication of likely ASD (at either invitation or enrollment) but who did not complete the study and to examine factors associated with inclusion in the final ASD case sample; (2) understand how the distributions of demographic and perinatal factors among mother–child pairs who completed the study and were classified as POP compared to mother–child pairs sampled from birth records and initially invited to potentially participate in SEED but who did not complete the study and to examine factors associated with inclusion in the final POP sample; (3) compare measures of association (odds ratios, ORs) for ASD-POP comparisons of sociodemographic and perinatal risk factors in (a) the final samples of ASD and POP children who completed the study and (b) a sample of likely ASD and POP children who were invited to potentially participate whether or not they enrolled or completed the study; and (4) derive post-stratification sampling weights for final ASD and POP participants and compare weighted and unweighted ORs for associations between ASD and select health factors.

### Data sources

The demographic and perinatal factors examined for study objectives 1 through 3 were child sex female, maternal age at birth > 35 years, 1 or more live births prior to index birth, maternal education at birth > 12 years, maternal race-ethnicity NOT non-Hispanic white, mother unmarried at birth, preterm delivery (< 37 weeks’ gestation), Cesarean delivery (either primary or secondary), and induction or stimulation of labor. All were ascertained from the birth certificate file.

For objective 4, we examined two maternal factors not available on the birth certificate: previous diagnosis of an infertility condition and reproductive stoppage after the index pregnancy. These two variables were selected for the current analysis because previous work by the authors examining these variables in the entire SEED sample indicated that the factor prevalence was sufficient to allow for relatively stable analyses in this single-site assessment. Additionally, earlier assessments had indicated that these factors were associated with ASD in opposite directions, which was of interest to this analysis of non-response impact.

Both infertility and reproductive stoppage were ascertained from the SEED computer-assisted maternal telephone interview. Infertility was derived from a question series in which mothers were asked whether before the index pregnancy a doctor or other healthcare provider ever told them it would be impossible or difficult to get pregnant—overall and because of specific infertility-related disorders, including blocked or damaged fallopian tubes; polycystic ovarian syndrome (PCOS) or multiple ovarian cysts; diminished ovarian reserve because of advanced age, premature ovarian failure or a medical condition; endometriosis; uterine problem, such as fibroids; or a diagnosis of unexplained infertility. Women could respond affirmatively to more than one disorder. Reproductive stoppage (no births after the index child’s birth) was derived from a question series in which mothers were asked for details of all pregnancies and births they had both before and after the index child.

### Study population

This analysis was limited to assessments of likely ASD and likely POP children born in singleton deliveries who were not missing birth certificate data for select demographic and perinatal factors included in this study (6% of subjects excluded due to missing values). Because the major research objectives of SEED involved comparison of ASD versus POP children on various risk factors and health conditions and because the invited DD group served a dual role—to serve as a secondary comparison group and to ascertain yet undiagnosed ASD case children—we did not include the DD group in this assessment. (However, as shown in Table 3 and described below, a sizable number of children identified for the potential DD group were yet included in this analysis because they screened positive for autism symptoms). As previously described, our final case–control classification was based on research-reliable assessments administered as part of the SEED research protocol. Therefore, at invitation or enrollment, we could not definitively determine what a child’s final case–control classification would be. Yet, the first three objectives necessitated definitions for likely ASD children and likely POP children among all invited children, regardless of whether they were contacted, enrolled, or completed the study. We used information from subjects who completed the study to estimate whether children in various initial study groups who did not enroll (for whatever reason) or who enrolled but did not complete the study would have likely been classified as cases or controls had they enrolled and completed the study.

**Table 3:** Final study analytic samples

ASD	POP
Likely ASD sample	Likely POP sample
N = 274 All children initially identified as presumptive ASD based on previous diagnosis of ASD—data from the subset who completed the study suggest an 84% likelihood of final classification ASD if non-responders/non-completers had enrolled and completed the study.	N = 2599 Children identified as presumptive POP from random sample of birth certificates minus the 9 children who had a positive autism screen at enrollment
N = 148 Children initially identified as potential DD or POP who were enrolled and had a positive autism screen—data from the subset who completed the study suggest a 51% likelihood of final classification ASD if non-completers had completed the study.	
Total N = 422	N = 2599
Final, complete ASD sample	Final, complete POP sample
N = 118	N = 159

Our sample of likely ASD children (n = 422) included (1) all children initially identified and invited for the ASD group; and (2) children initially identified and invited for the DD or POP groups who enrolled in the study and had positive ASD screens at enrollment. Our sample of likely POP children (n = 2599) included children initially identified and invited for the POP group with the exception of the small number who enrolled in the study and had positive ASD screens at enrollment. See Table 3 for a fuller description of children classified as likely ASD and likely POP. For the fourth objective, we used all of the data on invited participants to estimate invitation post-stratification sampling weights for the 277 children who completed the study and received a final classification of ASD case (n = 118) or POP control (n = 159).

### Data analysis

#### Objectives 1 and 2: factors associated with non-response among cases and controls

Within our samples of likely ASD children and likely POP children, we examined associations between inclusion in the final ASD sample or final POP sample and the aforementioned demographic and perinatal factors. We computed adjusted ORs and 95% confidence intervals (CIs) using logistic regression in which the outcome was inclusion in the final ASD sample or POP sample.

For both the ASD and POP assessments, sample size constraints precluded us from including specific maternal race-ethnicity subtypes in regression models. However, we separately examined the proportions of mothers who were non-Hispanic black and Hispanic and present relevant descriptive findings in the text.

#### Objective 3: impact of non-response on effect estimates

We examined whether differences between the final and invited case–control samples impacted measures of association. For both the final sample of cases and controls and the invited sample of likely cases and controls, we computed unadjusted and adjusted ORs for associations between ASD and each demographic and perinatal factor using logistic regression. Adjustment factors for each analysis included child sex, maternal age, maternal education, maternal race-ethnicity, and previous live births. We did not adjust ORs for perinatal factors because of the possibility of over-adjustment of some associations.

#### Objective 4: further assessment of non-response impact using sample weighting

To further explore whether measures of association for factors not available on the birth certificate were differential between the final and invited case–control samples, we created post-stratification sampling weights whereby we weighted the final sample to more closely match the initial invited sample. Post-stratification does not allow “zero” cells and works best with large samples. We were thus limited in the number of stratified variables and categories we could include. After checking cell sizes and associations with participation, we selected three variables for creating the weights: (1) initial classification at invitation based on data source and whether the child had documentation of ASD in school or clinic records (presumptive ASD, presumptive DD, presumptive POP); (2) maternal age at birth (≥ 30 or < 30 years); and (3) maternal education at birth (more than high school or high school or less). The maternal age and education cut points were determined empirically based on the cut points that best captured the variability in response rates rather than using other biologically- or sociologically-based definitions of interest for hypothesis-testing analyses.

We performed weighted and unweighted analyses to examine associations between ASD and previous diagnosis of an infertility condition and reproductive stoppage after the index pregnancy. Maternal infertility is of interest as a potential ASD risk factor. Reproductive stoppage is a potential health-related consequence of ASD. Thus, in the infertility analyses, ASD was the dependent variable and infertility was the independent variable and in the reproductive stoppage analyses, reproductive stoppage was the dependent variable and ASD was the independent variable. For both weighted and unweighted analyses, we computed ORs adjusted for child sex and maternal age, education, race-ethnicity, and previous live births using logistic regression.

In addition to analyses of maternal infertility and reproductive stoppage associations, we tested our weighting process by checking to see that associations with two demographic factors that had appeared biased in our initial assessment (see objective 3) were corrected when we applied the sampling weights.

Statistical analyses were conducted in SAS, version 9.3 (SAS Institute, Inc. Cary, North Carolina) and R version 3.3. GA SEED was approved by institutional review boards at the Centers for Disease Control and Prevention, Emory University, and the Georgia Department of Public Health.

## Results

### Objectives 1 and 2: factors associated with non-response among cases and controls (Table 4)

Mother–child pairs included in our final ASD case sample had fairly similar proportions of child sex female, advanced maternal age, previous live births, minority maternal race-ethnicity, unmarried at birth, preterm delivery, Cesarean delivery, and induction/stimulation of labor as those in the total sample of likely cases invited to participate in GA SEED. However, our final case sample included a higher proportion of mothers with advanced education at the time of birth (56.8%) than the invited case sample (45.5%). Moreover, after multivariable adjustment, the odds of maternal education > 12 years was 60% higher among those included than those not included in the final case sample.

**Table 4:** Socio-demographic and perinatal profiles of mother–child pairs in the final case and control samples and the initial samples of likely cases and controls invited to participate in GA SEED and assessment of factors associated with inclusion in the final samples

Factor	% Among final ASD cases who completed study (N = 118)	% Among all likely ASD cases invited (N = 422)	Adjusted OR^a^ inclusion in final ASD sample (95% CI)	% Among final POP controls who completed study (N = 159)	% Among all likely POP controls invited (N = 2599)	Adjusted OR^a^ inclusion in final POP sample (95% CI)
Child sex female	18.6	23.0	0.7 (0.4–1.2)	51.6	49.7	1.0 (0.8–1.4)
Maternal age > 35 years	26.3	21.1	1.4 (0.8–2.3)	23.3	13.7	*1.6 (1.1–2.4*)
Previous live births ≥ 1	46.6	52.4	0.8 (0.5–1.2)	52.2	58.7	0.7 (0.5–1.0)
Maternal education > 12 years	56.8	45.5	1.6 (1.0–2.5)	56.6	33.6	*2.2 (1.6–3.2*)
Maternal race/ethnicity NOT non-Hispanic white	51.7	58.5	0.8 (0.5–1.4)	45.9	61.9	0.7 (0.5–1.0)
Maternal marital status NOT married	61.0	67.5	0.7 (0.4–1.3)	65.4	70.2	1.0 (0.7–1.5)
Preterm delivery (< 37 weeks’ gestation)	16.1	15.9	1.2 (0.6–2.2)	10.7	9.9	1.3 (0.7–2.1)
Cesarean delivery (primary or repeat)	17.8	17.1	0.8 (0.4–1.6)	13.8	13.2	1.0 (0.6–1.6)
Induction/stimulation of labor	18.6	19.1	1.2 (0.6–2.3)	13.8	14.2	0.8 (0.5–1.4)

Data in italics indicate the confidence interval for the effect estimate does not include 1.0

^a^Adjusted models include all factors listed in the table

While there was little difference in minority race-ethnicity, overall, separate analyses indicated that even though the proportion of Hispanic mothers was generally low for the invited likely case sample, there was a marked difference in Hispanic proportion for the final (0.9%) versus invited sample (4.5%). This difference was not unexpected, given the English language eligibility requirement.

Mother–child pairs included in our final POP control sample had fairly similar proportions of child sex female, previous live births, unmarried at birth, preterm delivery, Cesarean delivery, and induction/stimulation of labor as those in the total sample of likely controls. However, the final control sample had notably higher proportions of maternal age > 35 years (23.3% vs. 13.7%), maternal education > 12 years (56.6% vs. 33.6%) and a notably lower proportion of minority race-ethnicity (45.9% vs. 61.9%). After adjustment, maternal age and maternal education were significant predictors of inclusion in the final control sample (ORs 1.6 and 2.2, respectively) but maternal race-ethnicity was not.

As was observed for the ASD case samples, separate analyses of race-ethnicity subgroups revealed a substantial variation in Hispanic proportion between the final and invited POP samples (0% vs. 15.6%).

### Objective 3: impact of non-response on effect estimates (Table 5)

In our final case–control sample, ASD was strongly associated with child sex (OR = 0.2 for female) and a similar-magnitude association was observed in the invited likely case–control sample (OR = 0.3). ASD was not associated with any of the other demographic or perinatal factors examined in the final sample; however, an association with preterm delivery was suggested, albeit with imprecise estimates (unadjusted OR = 1.6 [0.8–3.3]; adjusted OR = 1.8 [0.8–3.9]). In our invited likely case–control sample, ASD was also associated with preterm delivery; the effect estimates were very close to those observed in the final sample, but the CIs no longer overlapped 1.0 (unadjusted OR = 1.7 [1.3–2.3]; adjusted OR = 1.9 [1.4–2.5]). While the findings from the final sample indicated that ASD was not associated with either maternal age or education, findings from the invited sample indicated positive associations with both advanced maternal age (unadjusted OR = 1.7 [1.3–2.2]; adjusted OR = 1.7 [1.3–2.2]) and advanced maternal education (unadjusted OR = 1.7 [1.3–2.0]; adjusted OR = 1.7 [1.3–2.1]).

**Table 5:** Associations between ASD and socio-demographic and perinatal risk factors in the final sample of ASD cases and POP controls and the total invited sample of likely cases and controls

	Final complete sample ASD versus POP		Total invited sample Likely ASD versus Likely POP	
	Unadjusted OR (95% CI)	Adjusted OR^a^ (95% CI)	Unadjusted OR (95% CI)	Adjusted OR^a^ (95% CI)
Child sex female	*0.2 (0.1*–*0.4)*	*0.2 (0.1*–*0.4)*	*0.3 (0.2*–*0.4)*	*0.3 (0.2*–*0.4)*
Maternal age > 35 years	1.2 (0.7–2.0)	1.3 (0.7–2.4)	*1.7 (1.3*–*2.2)*	*1.7 (1.3*–*2.2)*
Previous live births ≥ 1	0.8 (0.5–1.3)	0.8 (0.5–1.3)	0.8 (0.6–1.0)	0.8 (0.6–1.0)
Maternal education > 12 years	1.0 (0.6–1.6)	1.0 (0.6–1.8)	*1.7 (1.3*–*2.0)*	*1.7 (1.3*–*2.1)*
Maternal race/ethnicity NOT non-Hispanic white	1.3 (0.8–2.0)	1.5 (0.8–2.5)	0.9 (0.7–1.1)	1.1 (0.8–1.3)
Maternal marital status NOT married	0.8 (0.5–1.4)	0.8 (0.4–1.3)	0.9 (0.7–1.1)	0.9 (0.8–1.2)
Preterm delivery (< 37 weeks’ gestation)	1.6 (0.8–3.2)	1.8 (0.8–3.9)	*1.7 (1.3*–*2.3)*	*1.9 (1.4*–*2.5)*
Cesarean delivery (primary or repeat)	1.3 (0.7–2.6)	1.1 (0.5–2.4)	1.3 (1.0–1.8)	1.3 (0.9–1.7)
Induction/stimulation of labor	1.4 (0.7–2.7)	1.4 (0.6–3.2)	1.0 (0.7–1.3)	0.9 (0.6–1.2)

Data in italics indicate the confidence interval for the effect estimate does not include 1.0

^a^For each factor, ORs were adjusted for the following factors if they were relevant as potential confounders (i.e. not the risk factor of interest): child sex, maternal age, previous live birth, maternal education, maternal race/ethnicity, and maternal marital status

### Objective 4: further assessment of non-response impact using sample weighting (Table 6)

After we applied post-stratification weights to our final case–control sample, we observed associations with both advanced maternal age and advanced maternal education that, although less precise, were similar to those observed in our analyses of the invited likely case–control sample (see Table 5). For maternal age, unadjusted and adjusted ORs from the weighted analyses were 1.7 [1.0–2.8] and 1.4 [0.7–2.8], respectively, and for maternal education unadjusted and adjusted ORs from the weighted analyses were 1.8 [1.2–2.6] and 1.9 [1.2–3.1]. These findings provide proof of concept that our weighting strategy was successful (i.e. that it reduced the bias due to factors known to be associated with participation).

**Table 6:** Unweighted and weighted analyses of associations between ASD and select maternal factors

	Unweighted final sample ASD versus POP		Weighted final sample ASD versus POP	
	Unadjusted OR (95% CI)	Adjusted OR^a^ (95% CI)	Unadjusted OR (95% CI)	Adjusted OR^a^ (95% CI)
Maternal infertility	1.8 (0.9–3.7)	1.9 (0.9–3.9)	*2.5 (1.1*–*5.8)*	2.2 (0.8–5.8)
Reproductive stoppage	0.6 (0.4–1.0)	0.6 (0.4–1.1)	0.6 (0.3–1.0)	0.8 (0.4–1.5)

Data in italic indicates the confidence interval for the effect estimate does not include 1.0

^a^ORs were adjusted for child sex, maternal age, previous live birth, maternal education, maternal race/ethnicity, and maternal marital status

The findings from our unweighted and weighed analyses of associations between ASD and two reproductive history factors were similar. Estimates from both sets of analyses were imprecise as evidenced by fairly wide CIs, most of which overlapped 1.0. Nonetheless, the point estimates for the ASD-maternal infertility assessment indicated a moderate positive association in both unweighted and weighted samples. The point estimates for the ASD-reproductive stoppage assessment indicated an inverse association in both samples.

## Discussion

Altogether these findings indicate that two factors—maternal age and education—were independently associated with participation in the GA SEED POP control group and one of these factors, maternal education, was also independently associated with participation in the GA SEED case group. Also, we cannot rule out an association between maternal age and participation in the case group because our sample size was fairly small for this group and the adjusted OR, while not significant, was moderately elevated (1.4). These participation effects impacted hypothesis-testing analyses of associations between ASD and maternal age and education. Using the final GA SEED sample to assess associations between ASD and these two factors would lead to an erroneous interpretation. However, our findings were reassuring overall, in that effect estimates from analyses of associations of several other demographic factors, several perinatal factors, and maternal reproductive history factors were not impacted by low study participation. Thus, the findings from other SEED risk factor analyses, particularly those of non-demographic factors, are likely robust.

Beyond SEED, our findings are potentially informative to other population-based case–control studies. Our analyses provide empiric support that studies with expansive recruitment strategies to include a wide segment of the population can provide valid data on many condition-risk factor associations even if invited participants are difficult to locate and contact. Specifically, as demonstrated by others, a complete-records analysis leads to unbiased associations for many exposure variables, provided study participation is not associated with both the independent variable of interest and the outcome [2, 4]. Additionally, analogous to our findings, Aigner et al. [21] demonstrated that it is possible to produce unbiased effect measures in case–control studies with differential response rates, so long as the covariates associated with response rates are included in the multivariable logistic regression model or used to inform inverse probability weights. They also found that simulations to estimate the magnitude of the bias of missingness might be useful.

This is critically important for conditions, such as ASD, for which the epidemiologic literature has only recently begun to evolve. In fact, it is difficult to compare our data on participation in SEED with those from other ASD studies. The vast majority of studies worldwide that examine ASD risk factors have used existing health and administrative databases—for example, large health registry linkages in Scandinavian countries and health claims data or health maintenance organization data from select practices in the United States. While these data have been valuable in informing ASD risks, they are limited in both case and exposure ascertainment. Case ascertainment is not standardized and is subject to varying levels of potential classification bias since many children with ASD (particularly those from minority and low socio-economic status families) have delayed diagnosis and will not be included in existing databases; moreover, existing databases of ASD lack critical information to construct important case subgroups. In contrast, SEED includes a diverse population-based sample of children with ASD with heterogeneous and robustly characterized phenotypes. Exposure/risk factor data from existing data sources are also limited; many important exposures are not included at all and many important details are lacking for exposures that are included. SEED was specifically designed to address these limitations.

To our knowledge there is only one study designed in a similar manner to SEED—the California Childhood Autism Risks from Genetics and the Environment (CHARGE) study. The CHARGE study utilized recruitment and data collection methods similar to SEED; however, the CHARGE sample is much smaller—only 25% that of SEED sample (pooled sample from all six SEED sites). While the CHARGE study researchers have not provided data on the proportion of invited participants who were not contacted, the researchers present the following on post-contact data: “Among contacted families of children with autism, 20% were ineligible, 22% refused and 58% agreed to participate. Among general population families with whom we made contact, 22% were ineligible, 41% refused and 36% agreed to join the study” 22]. These eligibility and enrollment rates are generally in line with those for SEED. Across SEED sites, 22% of the potential ASD or DD families who were contacted were found to be ineligible, 34% refused participation before eligibility could be determined, and 43% were eligible and enrolled. Among potential POP families contacted, 34% were known to be ineligible, 40% refused before eligibility could be determined, and 25% were eligible and enrolled.

Our study has many strengths. Most notably, we had data on the distribution of various characteristics among all individuals invited to GA SEED. While this is highly recommended for all epidemiologic studies such that researchers are able to incorporate assessments of potential non-response impacts into their analyses [21], many studies are not able to obtain such information. We used our data on non-responders both to study the impacts of non-response directly and to derive post-stratification sampling weights such that we could extend our evaluation to factors not available on the birth certificate, but only collected on our final sample.

Despite the study strengths, the findings must also be interpreted in the context of limitations. Most notably, because only one of the SEED sites was able to ascertain information on non-participants, the sample size was small and many of the findings reported here were imprecise. Nonetheless, our findings from different types of response impact assessments yielded consistent findings, indicating convergent validity. Sample size constraints also limited our unweighted and weighted analyses of the final case–control sample, in that we could only examine factors that had a fairly high population prevalence. Moreover, the small sample sizes for some analyses limited the precision of the effect measures observed. Thus, while here we generally assess the impact of non-response on measures of association with the aim of discerning whether non-response effects might have led us to an incorrect conclusion, we did not attempt to specifically quantify the magnitude of possible non-response effects. We also could not empirically assess the generalizability of our findings to other SEED sites; however, all sites followed a common recruitment and data collection protocol and encountered similar issues as GA SEED in contacting invited participants. Nonetheless, during the time of this study the proportion of children in the GA SEED study who were Hispanic was fairly low; thus, the GA SEED site did not include Spanish language materials and as a result, our final sample included a very low number of Hispanic children. Hence, we could not adequately examine the impact of Hispanic ethnicity on study participation; nor could we consider Hispanic ethnicity as a potential confounder in adjusted risk factor analyses presented here. Therefore, our data may not be entirely generalizable to other SEED sites with higher proportions of Hispanic children. While strengths of this study are comprehensive case-finding, including identifying cases from young children without a previous ASD diagnosis and rigorous research-reliable case classification methods, these features made it difficult to cleanly define total invited case and control groups with certainty. Thus, it is likely that some of the children included in our invited samples would not have received a final study classification of case or control that matched the invited sample in which we placed them had they completed the study. Most notably, as shown in Table 3, our likely ASD case sample is comprised of two general subgroups of children: children with a previous (community) ASD diagnosis (65% of the total likely ASD sample) and children initially identified for the DD or POP group who had a positive autism screen at enrollment (35% of the sample). We did not attempt to parse our analysis into separate assessments of these subgroups because neither of these on their own match our final case sample. Of the 118 children in our final ASD case sample, 54% had a previous ASD diagnosis and 46% were initially identified through the study autism screen.

## Conclusion

Using GA SEED data, we demonstrated empirically that while select demographic factors were directly associated with participation in a population-based ASD case–control study, other demographic and biologic factors were not. Moreover, we demonstrated that analyses of associations of biologic factors—both perinatal factors on the birth certificate and reproductive health history factors captured via maternal interview—were not impacted by low participation rates. Additionally, even though differential participation by some population subgroups limited our ability to examine associations between ASD and two demographic factors—maternal age and education—this study demonstrated that the effect estimates for associations with several other demographic factors were not patently biased. SEED is an important source of information on ASD risk factors. Although, it is important to carefully consider each SEED analysis individually in terms of whether the risk factor or health outcome of interest might be associated with study participation, this study generally lends support to the robustness of findings already generated or soon to be generated from SEED.

## Abbreviations

ASD: autism spectrum disorder
DD: developmental disabilities
GA SEED: Georgia Study to Explore Early Development
PCOS: polycystic ovarian syndrome
POP: population controls
SCQ: Social Communication Questionnaire
SEED: Study to Explore Early Development
